# Slug and Vimentin downregulation at the metastatic site is associated with Skip-N2 metastasis of lung adenocarcinoma

**DOI:** 10.1007/s12672-022-00467-4

**Published:** 2022-01-22

**Authors:** Yasemin Saygideger, Alper Avci, Emine Bagir, Burcu Saygıdeğer Demir, Aycan Sezan, Mucahit Ekici, Oya Baydar, Özgür Cem Erkin

**Affiliations:** 1 Department of Pulmonary, Cukurova University School of Medicine, Adana, Turkey; 2 Institute of Health Sciences, Department of Translational Medicine, Cukurova University, Adana, Turkey; 3 Department of Thoracic Surgery, Cukurova University School of Medicine, Adana, Turkey; 4 Department of Pathology, School of Medicine, Cukurova University, Adana, Turkey; 5 Department of Biotechnology, Institute of Natural and Applied Sciences, Cukurova University, Adana, Turkey; 6 Department of Bioengineering, Faculty of Engineering, Adana Alparslan Türkeş Science and Technology University, Adana, Turkey

**Keywords:** Lung cancer, Skip N2, EMT, SLUG, TP63

## Abstract

**Objective:**

Lung cancer displays heterogeneity both in the tumor itself and in its metastatic regions. One interesting behavior of the tumor is known as Skip N2 metastasis, which N2 lymph nodes contain tumor cells while N1 are clean. In this study, mRNA levels of epithelial mesenchymal transition (EMT) related genes in skip N2 and normal N2 involvements of non-small cell lung cancer tissues were investigated to evaluate the possible molecular background that may contribute to the pathogenesis of Skip N2 metastasis.

**Materials and methods:**

Eighty-three surgically resected and paraffin embedded lymph node samples of lung cancer patients were analyzed in this study, which 40 of them were Skip N2. N2 tissues were sampled from 50% tumor containing areas and total RNA was extracted. mRNA levels for 18S, E-cadherin, Vimentin, ZEB1 and SLUG were analyzed via qPCR and E-cadherin and vimentin protein levels via immunohistochemistry (IHC). Bioinformatic analysis were adopted using online datasets to evaluate significantly co-expressed genes with SLUG in lung cancer tissue samples.

**Results:**

Skip-N2 patients who had adenocarcinoma subtype had better survival rates. Comparative analysis of PCR results indicated that Skip N2 tumor tissues had increased E-Cadherin/Vimentin ratio and ZEB1 mRNA expression, and significantly decreased levels of SLUG. E-cadherin IHC staining were higher in Skip N2 and Vimentin were in Non-Skip N2. TP63 had a strong correlation with SLUG expression in the bioinformatics analyses.

**Conclusion:**

The results indicate that, at molecular level, Skip N2 pathogenesis has different molecular background and regulation of SLUG expression may orchestrate the process.

## Highlights

- Skip N2 metastasis is related with better on-year survival rates in Lung Adenocarcinoma
- EMT regulator gene *SLUG* expression is suppressed in Skip N2 compared to Non-Skip N2 metastasis.
- Tumor metastatic site exhibit less Vimentin staining at Skip N2 lymph node
- Bioinformatic analyzes show that one of the genes with the highest expression correlation with SLUG was TP63, suggesting that they may be acting together in this process.

## Introduction

Lung Cancer, which ranks first in cancer-related deaths in the world, is being investigated intensively with studies based on advances in molecular biology and technology. Lung cancers generally show intra-tumoral heterogeneity and use adaptation and resistance mechanisms extensively. In this case, targeted therapies and close monitoring and understanding of the adaptive response and resistance mechanisms developed by the tumor play an important role in the fight against this cancer. Development of targeted therapies is possible by getting to know the biological behavior of cancers closely.

Lung cancers are histologically divided into 2 main subtypes: Non-Small Cell Lung Cancers (NSCLC) and neuroendocrine tumors. NSCLC staging is made according to the TNM staging system. Here, T = tumor size, N = lymph node involvement and M = metastasis. Lymph node metastasis is important in lung cancer staging. In summary, N1: Involvement in the same sided peri bronchial and hilar lymph nodes, N2: Involvement in the mediastinal or subcarinal lymph nodes on the same side, N3 refers to involvement in the contralateral mediastinal, hilar, or the same/opposite sided scalene or supraclavicular lymph nodes [1].

While lymph node involvements are expected to follow a sequential path depending on the lymphatic spread of the tumor, it is observed that N2 nodes are involved in some cases without N1 involvement. This type of involvement has been termed pathological N2 or Skip N2. Adenocarcinoma, the most common subtype of NSCLC, is the histological subtype most associated with Skip N2 [2]. Studies have reported that the behavior and profiles of Skip N2 and normal N2 lung cancers, their response to treatment and the genetic mutations they show are different from each other [3]. The mechanism of Skip N2 involvement is still not fully understood.

Epithelial-mesenchymal transition (EMT) is the transformation of epithelial tumors into a more durable and more suitable for metastasis due to the genetic and morphological changes they undergo as a result of their response to stress and other stimuli. It has been experimentally demonstrated that in many cancer cell-lines, epithelial cells stimulated with TGF-Beta take on a spindle morphological appearance, increase their invasion and metastasis properties, gain stem cell character and play a role in treatment resistance. Epithelial tumor is expected to pass through the mesenchymal-like state in order to metastasize and regain its epithelial properties after metastasis [4, 5]. Increase in the expression levels of EMT related genes in lung cancer lymph nodes, were found to be associated with poor prognosis [6]. But the role of EMT process in skip metastasis remains unknown.

In this study, we aimed to evaluate the expression levels of epithelial cell marker E-Cadherin and mesenchymal cell markers Vimentin, ZEB1 and SNAI2 (SLUG) mRNA levels in surgically resected and paraffin embedded lymph node samples of lung cancer patients and to compare the overall survival rates of the lung cancer patients with Skip N2 and normal N2 metastasis.

## Materials and methods

### Patients and samples

The study was carried out at a single center university hospital. Patients’ records, who had surgical operation in between 2012 and 2016 due to lung cancer were retrospectively reviewed for their lymph node metastasis. The study was performed in accordance with the latest ethical standards of Helsinki Declaration and approved by Cukurova University’s Ethical Board (2019/84-40). N2 positive patients were included in the study and paraffin tissues for N1 and N2 were re-examined by the pathologist. Skip N2 and normal N2 involvement in lymph node examinations. Tissue samples were sliced from at least 50% tumor containing areas.

### mRNA isolation, cDNA synthesis and Quantitative Polymerase Chain Reaction (qPCR)

Total RNA was extracted from paraffin tissues using FFPE-RNA isolation kit (RTA) and reverse transcribed using cDNA reverse transcription kit (Thermo Fisher) according to manufacturer’s protocols. Quantitative PCR was done using SYBR Green mix (Sigma Aldrich). Reactions were performed in triplicate in a 96-multiwell plate (Roche). Gene expressions were normalized to *18S rRNA*, and fold differences were calculated using the comparative CT method: 2-(ΔΔCT), where ΔΔCT refers to (normalized control sample) − (normalized treated sample) as previously described [6].

### Primers

Primers were obtained using the IDT DNA PrimerQuest Tool [7], after retrieving the nucleotide sequences of the genes in FATSA format from the Pubmed NCBI GENE. The selection of the obtained primers was made by using the Primer-BLAST computer program [8], after investigating their specificity. Primer pairs were as follows; *18S rRNA*: sense: 5′-cttagagggacaagtggcg-3′, antisense: 5′-acgctgagccagtcagtgta-3′; *VIM*: sense: 5′-gagaactttgccgttgaagc-3′, antisense: 5′-gcttcctgtaggtggcaatc-3′; *ECAD*: sense: 5′-acactgccaactggctggagatta-3′, antisense: 5′-tgattagggctgtgtacgtgctgt-3′; *ZEB1*: sense: 5′-cttctcacactctgggtcttattc-3′, antisense: 5′cgttcttccgcttctctcttac-3′; *SNAI2 (SLUG)*: sense: 5′-gcgatgcccagtctagaaa-3′, antisense: 5′-ggtaatgtgtgggtccgaata-3′.

### Immunohistochemistry

Immunohistochemical staining was performed on 5–μm sections of formalin-fixed, paraffin-embedded tissue using antibody to Vimentin (NCL-L-VIM-V9; Novocasrtra, UK) and E-Cadherin (NCH-38; Dako, CA USA). The visualization system used was BenchMark XT with heat-induced epitope retrieval (CC1 solution, Ventana) and iView DAB detection kit (Ventana, Tucson, AZ).

### Bioinformatic analysis

Freely available patient derived tumor array databases and web-based online tools were used for bioinformatics analyses. Five different Oncomine Datasets that consists of lung cancer tissue samples and online analysis tools (https://www.oncomine.org/resource/main.html) were used for co-expression analyses [9–12] and the cancer genome atlas (TCGA) lung cancer datasets were analyzed by GEPIA (http://gepia.cancer-pku.cn/) for correlation calculations [13].

### Statistical analysis

The statistical significance of differences between groups was evaluated by an unpaired Student t tests and chi-square tests, and survival statistics were determined via Mantel-Cox test using Prism 9 (GraphPad Software, Inc.).

## Results

### Demographic features of the patients were similar in between Skip N2 and Non-Skip N2 groups

A total of 250 records were reviewed and 83 patients were included in the study. The patients who did not have N2 tumor involvement, who had N3 involvement or died due to the complications of the surgery are excluded from the study (n = 135). The extracted RNA yield was suitable for 40 of 48 Skip N2 positive and 43 of 67 Normal N2 (Non-Skip) samples. There were no significant differences in age, gender, pathological subtype of the disease, localization and size of the tumor as well as overall survival rates and lymph node SUVmax ratios between the groups (Table 1). We would like to note that the differences in the treatment regimens and the mortality due to chemo or radiotherapy are not considered during our survival analyses.

**Table 1:** Comparison of the clinical parameters of Skip and Non-Skip N2 patients

	Skip N2 (Pathological N2) n = 40	Non-Skip N2 (Normal N2) n = 43	Significance
Gender	M = 35 (87.5%) F = 5 (12.5%)	M = 35 (83.3%) F = 8 (16.7%)	p = 0.5513
Age	58.125 (± 9.25)	60.95(± 10.42)	p = 0.1987
Pathology	Adenocarcinoma: 16 (40%) Squamous cell: 18 (45%) Others: 6 (15%)	Adenocarcinoma: 26 (62%) Squamous cell: 15 (37.5%) Others: 2 (0.5%)	p = 0.1555
Tumor localization	Right lung: 21 Left lung: 19	Right lung: 24 Left lung: 19	p = 0.7620
	Superior lobes: 25 Middle-inferior lobes: 15	Superior lobes: 23 Middle-inferior lobes: 20	p = 0.4061
T factor	T1: 13 T2: 17 T3: 7 T4: 3	T1: 14 T2: 15 T3: 10 T4: 4	p = 0.6638
Mediastinal lymph node SUV	19 Negatives	17 Negatives	p = 0.4644

### One-Year survival rate was significantly increased in Skip N2 Adenocarcinoma patients

**Fig. 1:**
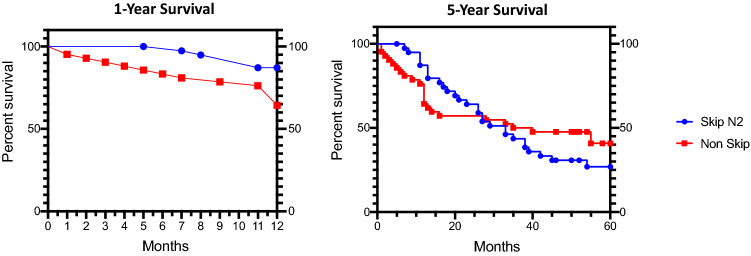
Percent survival rates for 12 and 60 months are given in the Kaplan–Meier Curves for Skip-N2 and Non-Skip N2 patients

**Fig. 2:**
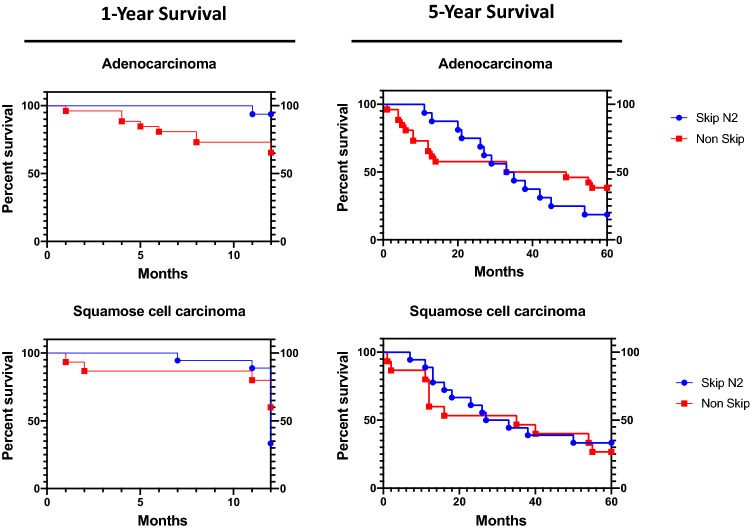
Percent survival rates for 12 and 60 months in Adenocarcinoma and Squamous Cell Carcinoma histologic subtypes

Since the overall survival rates between the two groups were similar, we performed both 60 and 12-months survival graphs to visualize the differences in the survival rates between the two groups. According to the survival analysis, results for 5-year favored Normal N2 comparing to Skip N2 group which the median survival rates were 33 months and 37 months for Skip N2 and Non-Skip N2 respectively, but the difference was not statistically significant (p = 0.51) (Fig. 1, right panel). The 1-year survival analyzes resulted in favor of Skip N2 patients, in contrast to 5-year results, and the difference was statistically significant (p = 0.0157) (Fig. 1, left panel). We than, performed survival analyses by dividing the patients into two groups according to their histological subtype. The only difference between Adenocarcinoma (AC) and Squamous-Cell Carcinoma (SC) groups was the localization of tumor which, adenocarcinomas localized mainly at the superior lobes (Table 2). In the 5-year analyses, survival rates for Skip and Non-Skip N2’s were similar in both AC and SC (p = 0.473 and p = 0.659 respectively), but the 1-year results revealed that Skip N2 Adenocarcinoma patients had better survival rates comparing to Non-Skip group (p = 0.038) SC group did not have the similar outcome (p = 0.310) (Fig. 2).

**Table 2:** Comparison of Skip N2 patient characteristics in Adenocarcinoma and Squamose Cell Carcinoma patients

	Adenocarcinoma n = 16	Squamose Cell Carcinoma n = 18	Significance (*p < 0.05)
Gender	M = 15 (93.75%) F = 1 (6.25%)	M = 18 (100%) F = 0 (0%)	p = 0.4706
Age	57.825 (± 9.86)	60.94 (± 7.58)	p = 0.3046

Tumor localization	Superior lobes: 14 Middle-inferior lobes: 2	Superior lobes: 8 Middle-inferior lobes: 10	p = 0.0129*
T factor	T1: 6 T2: 7 T3: 2 T4: 1	T1: 5 T2: 8 T3: 3 T4: 2	p = 0.3910
Mediastinal lymph node suv	7 Negatives (43.75%)	10 Negatives (55%)	p = 0.4920

### Vimentin and SLUG mRNA levels were significantly downregulated in Skip N2 samples

**Fig. 3:**
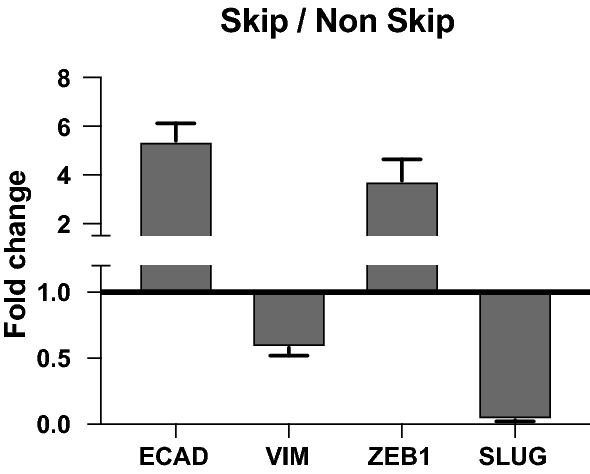
Quantitative PCR results presenting mRNA levels of EMT related genes. The results represent the average expressions of the studied genes. 18S was used as internal control gene

In order to determine the expression profile of EMT related genes, we extracted total RNA from paraffin embedded tissue samples. All samples underwent for RNA extraction, and the amount of extracted RNA was 79.71 $\pm$ 72 ng/μL and complementary DNA (cDNA) was synthetized before quantitative PCR (qPCR) experiment. We then performed qPCR analysis to evaluate mRNA levels of epithelial to mesenchymal transition (EMT) related genes *ECAD*, *VIM*, *ZEB1* and *SNAI2* (*SLUG*). Cycle threshold (CT) values of each gene were normalized to internal control gene *18S,* and to calculate the fold differences between the two groups comparative CT method was used as described in the methods section. The overall results revealed that epithelial marker E-cadherin mRNA levels, were increased and mesenchymal marker vimentin were decreased in Skip N2 lymph node samples comparing to Non-Skip N2 (Fig. 3). Moreover, while ZEB1 was found increased, SLUG mRNA levels were significantly decreased in Skip N2 samples, suggesting a possible molecular action of these genes may play role during the pathogenesis of Skip N2 metastasis of lung cancer.

### Skip N2 tissue IHC staining showed increased E-cadherin expression

**Fig. 4:**
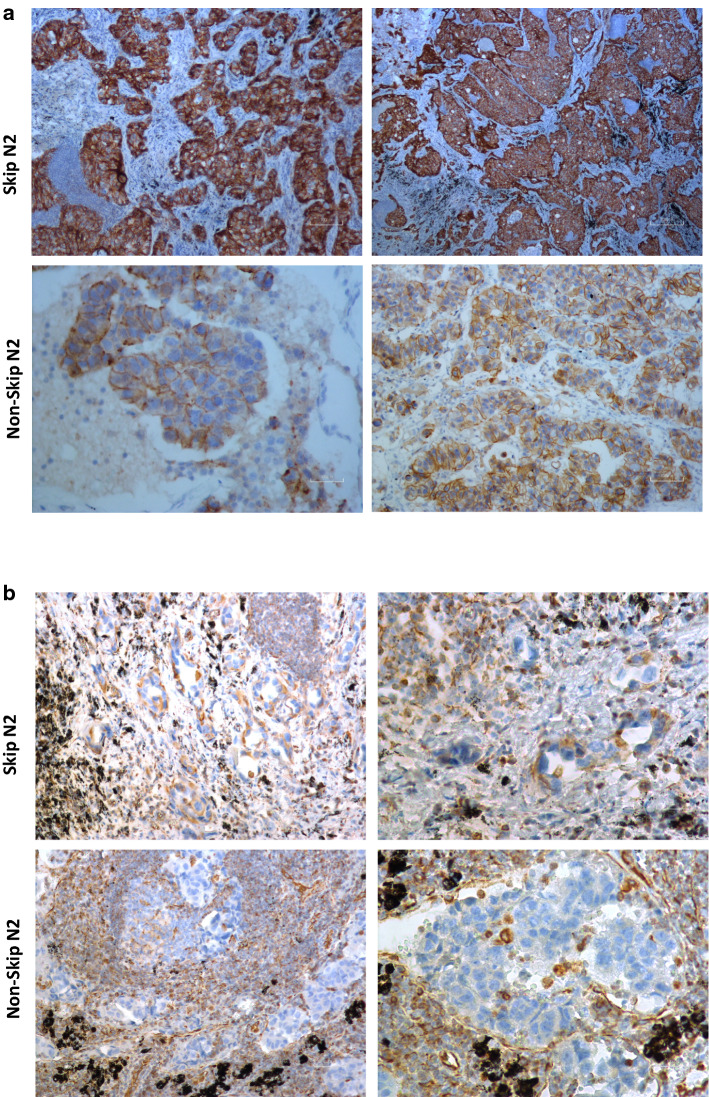
Immunohistochemistry staining via E-cadherin and Vimentin in lung adenocarcinoma metastasis of N2 lymph nodes. **a** Skip N2 samples from two different patients exhibit increased E-cadherin expression comparing to two Non-Skip N2 samples. **b** Vimentin expression is likely to surround tumor cells in both Skip and Non-Skip N2 tissues, while Non-Skip samples had increased Vimentin staining comparing to Skip N2

We further performed IHC analysis in two Skip N2—Adenocarcinoma and two Non-Skip N2-Adenocarcinoma samples using E-cadherin and Vimentin antibodies in order to confirm our PCR results. The staining with E-cadherin antibody was higher in Skip N2 samples comparing to Non-Skip N2 in most of the collected images (Fig. 4a). As expected, Vimentin levels were too low in the tumor area, but localized around the metastatic site in all the samples studied and the staining was somewhat increased in Non-Skip N2 samples (Fig. 4b). These findings supported the results obtained via PCR experiments.

### SLUG expression is strongly correlated with apoptosis regulator protein TP63

**Fig. 5:**
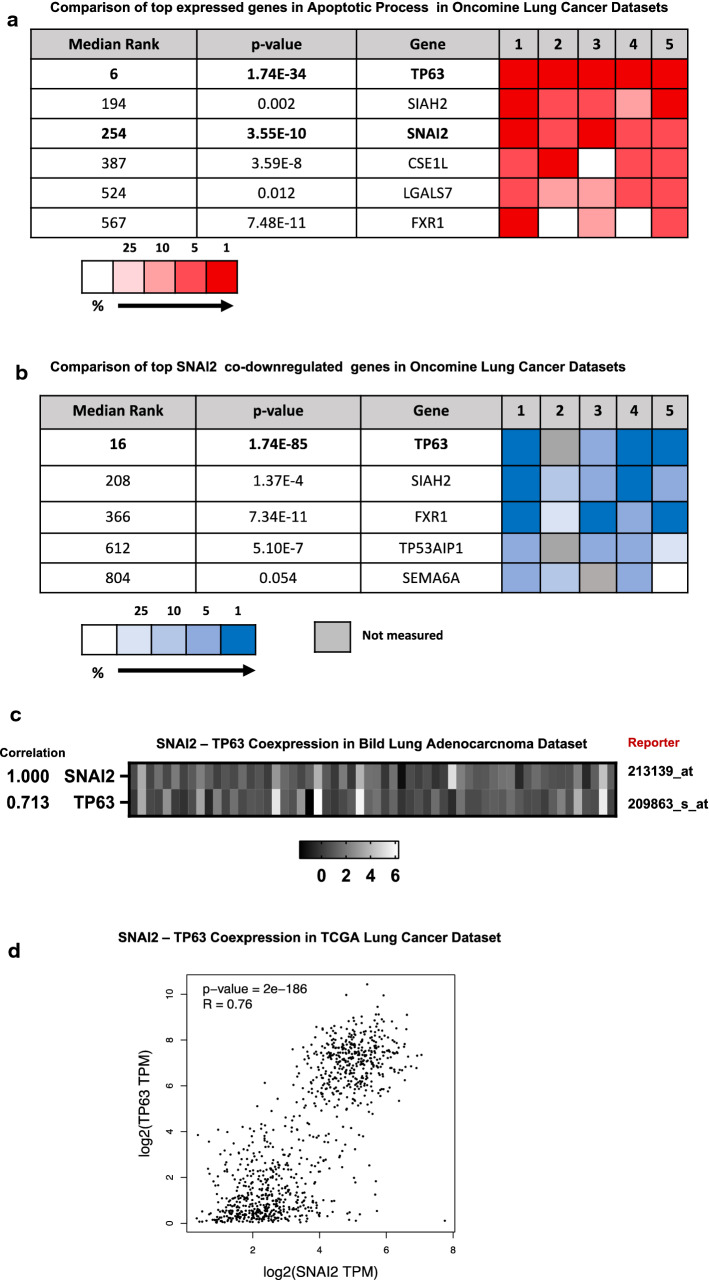
Bioinformatic analyses of SNAI2 and TP63 expression in lung cancer tissue samples. **a** Heatmap of genes that are top co-expressed in NSCLC tumor samples in apoptosis concept (Gene Ontology Biological Process) in 5 different Oncomine datasets consisting of 470 patient samples. The rank for a gene is the median rank for that gene across each of the analyses. The p-Value for a gene is its p-Value for the median-ranked analysis. **b** Comparison of top SNAI2 co-downregulated genes in the same datasets with A. **c** Correlation of SNAI2 and TP63 expression in Lung Adenocarcinoma tissue samples in Oncomine *Bild Lung* dataset (n = 58). **d** Correlation of SNAI2 and TP63 mRNA levels in TCGA lung cancer datasets in 969 patients

According to the PCR results, since the gene with the most different mRNA expression between the two groups was SLUG, the analyzes of other genes likely to play a role together with SLUG were analyzed by bioinformatics tools. Using Oncomine analysis tool, which is an online discovery platform that provide transcriptomic database of various cancers to the researchers, we selected 4 datasets (Bild Lung, Raponi Lung, Rohrbeck Lung and TCGA Lung) consisting of 470 clinical specimens obtained from lung cancer patients to evaluate mRNA levels of top 1% co-expressed genes. The co-expression analyze threshold values were set as p value to 1e-8 and fold change to 2, and we specifically used apoptosis concept (Gene Ontology Biological Process) due to the absence of tumor metastasis in N1 nodes. According to the results, mRNA levels of tumor protein 63 (TP63), which is a member of p53 transcriptor family, had the highest rank and SNAI2 was at the third row in top 1% in apoptosis concept related co-expressed genes (Fig. 5a). Using the same datasets and concept, we also analyzed top 1% SNAI2 co-expressed genes and interestingly, TP63 was at the top of the under-expression heatmap (Fig. 5b) but did not rank in over-expression list (data not shown), suggesting that expression of these two genes might be related to each other or affected by the similar signaling pathways. Therefore, we analyzed correlation of co-expression of these two genes in Bild Lung dataset of 58 lung adenocarcinoma samples and generated a heatmap using Oncomine analysis tool. The results indicated a strong correlation in the expression of these two genes in the selected dataset (Fig. 5c). We further analyzed TCGA lung cancer datasets consisting of 483 lung adeno-cancer and 486 lung squamous cell carcinoma tumor samples to determine expression correlation of SLUG and TP53 mRNA levels using GEPIA correlation analyses tool [13], and Spearman’s correlation coefficient. The results indicated a strong correlation (R = 0.76, p = 2e−186), (Fig. 5d).

## Discussion

**Fig. 6:**
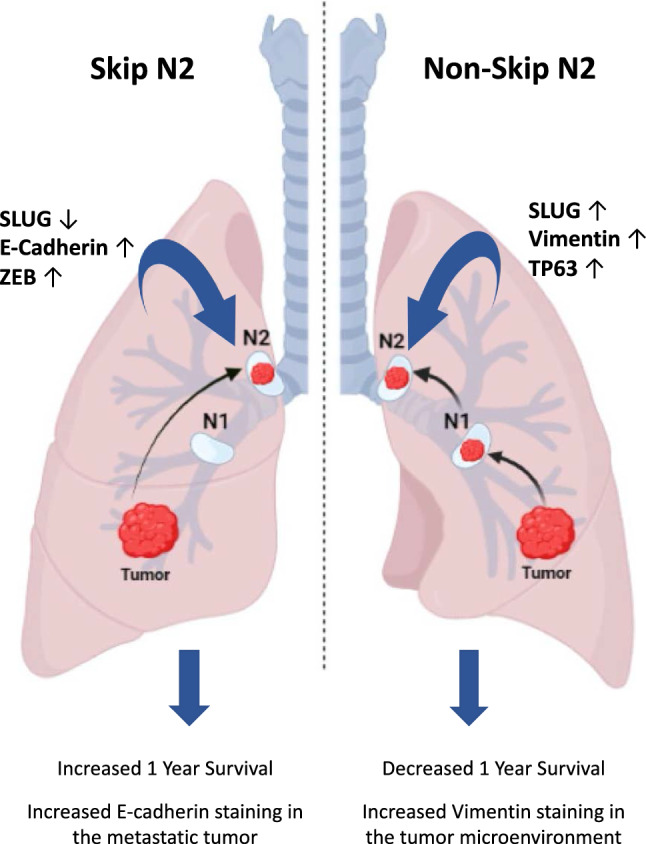
Overall summary of the results and proposed molecular background that contribute to lymph node metastasis

In this study, mRNA levels of EMT-related genes *ECAD*, *VIM*, *ZEB1*, *SLUG* was evaluated in Skip N2 metastasis, which is a common entity in the lymphatic spread of the tumor in lung cancer patients and the molecular mechanism has not yet been elucidated. The findings of the study showed that tumors with Skip N2 features were associated with better prognosis, especially in the histological subtype of Adenocarcinoma, at 1-year survival compared to tumors with sequential N1 + N2 metastasis. These findings were also supported by examining mRNAs expressions isolated from paraffin blocks of patients’ lymph node by qPCR method. According to these analyzes, the epithelial marker E-cadherin, which is a better differentiation marker, was found in high rates in Skip N2 tissues, while SLUG and parallel to this, Vimentin mRNA expressions were suppressed compared to Non-Skip N2 tissues. A moderate increase in ZEB1 levels, which is also known as an EMT factor, in Skip N2 tissues in contrast to SLUG was noted. Overall summary of the findings of this study is illustrated in Fig. 6.

Slug is a protein that belong to the SNAIL family transcription factors, and it contributes significantly to the EMT process and therefore, plays a role in the metastasis of cancer cells, by reducing the expression of the E-cadherin protein, which provides intercellular connectivity [14]. Slug expression is normally at very low levels in epithelial cells and therefore its increase is associated with poor differentiation and thus poor prognosis. In this study, the non-Skip N2 group with high Slug expression was found to have a worse 1-year survival compared to the Skip-N2 group with significantly suppressed Slug levels. The disappearance of this difference in 5-year survival may be related to the selective tumoral cloning or intra-tumoral heterogeneity, frequently encountered in lung cancer, in particularly adenocarcinoma subtype [15].

There are similar studies in the literature that find that Skip N2 metastasis is associated with overall survival in lung cancer [16–18]. One of these studies reported that Skip N2 is a good prognostic indicator when it is involved in the upper lobes, especially in Adenocarcinomas [16], even though many other studies have found it predominantly in the upper lobe, it has indicated that no statistical significance was found in this difference [17]. According to our findings, while neither tumor size, histological subtype, nor other demographic factors were associated with overall survival, 1-year survival was found to be a good prognostic indicator, especially in adenocarcinomas. According to the International Association for the Study of Lung Cancer Staging Project, it has been recommended that the physicians should clearly record the pathological N2 presence and detailed anatomical features of the lymph node metastasis status of the patients. They have pointed out that it might be a strong possibility to be included in the classification as an indicator [19–21]. Moreover, a recently published meta-analysis study including 29 articles revealed that the presence of Skip N2 is a good prognostic indicator [22].

Studies to elucidate the molecular mechanism of Skip N2 are very limited in the literature. In two studies, epidermal growth factor receptor mutation was found to be higher in patients with Skip N2, but both could not suggest a mechanism for the possible contribution of tyrosine kinase receptors to the pathogenesis [3, 22]. In a study investigating the relationship between tumor long diameter and Ki67 expression levels, which is a proliferation marker, with Suv_max_ in lung cancer, it was shown that Ki67 was higher in the presence of Skip N2, but this was not statistically significant due to the insufficient number of samples [23]. In this study, Skip N2 encodes a protein that plays an important role in EMT, such as ZEB1, Slug, in which different mRNA expression such as SLUG is detected in the examinations performed on metastatic tissues. In the studies conducted, while the factors that contribute to both ZEB1 and SLUG EMT and cause the suppression of E-Cadherin expression, the intracellular targets of both differ from each other. For example, while ZEB1 plays a more effective role in the change of epithelial cell polarity and promotion of proliferation, Slug stands out with its suppression of Claudin-1 expression and its role in the development of resistance to apoptosis (24). In the literature, where the differences and additional functions of proteins involved in EMT were compiled, it was reported that Slug expression increased in hypoxic lung adenocarcinoma tissue, but ZEB1 expression did not change [24, 25]. It has been reported that Fragile Histidine Triad (FHIT) pathway disorder, which is known to play a role in Cisplatin resistance and is frequently reported to be inactivated in lung cancer, especially in smokers, increases cell invasion through Slug, but a similar interest has not yet been observed with ZEB1 [26]. On the other hand, there are studies showing that the expression of these two proteins, which work in the same pathway, is regulated by different microRNAs in the translational process. For example, while miR-218 suppresses protein levels by targeting SLUG and ZEB2 in lung cancer, it does not have such an effect on ZEB1 [27]. In addition, in a study conducted in head and neck squamous cell carcinoma, it was shown that the ΔNp63 isoform of the p63 protein encoded by the TP63 gene is necessary and sufficient for Slug-mediated EMT activation [28]. Bioinformatic analyzes of our study revealed a significant correlation between TP63 expression and SLUG expression in non-small cell lung cancer samples, suggesting that the two proteins act together in the EMT process. Recent studies also indicate that ΔNp63 isoform of p63, also regulates collective invasion package (CIP) during lung cancer metastasis [29], and Vimentin is required for CIP, which is also poor prognostic indicator in lung adenocarcinoma cells [30], compatible with our findings in this research.

This research also indicated genes other than TP63 that were co-downregulated with SNAI2 in several online Oncomine Lung Cancer datasets. These were SIAH2 (Siah E3 Ubiquitin Protein Ligase 2), FXR1 (Fragile X Mental Retardation Syndrome—Related Protein 1), TP53AIP1 (Tumor Protein P53 Regulated Apoptosis Inducing Protein 1) and SEMA6A (Transmembrane semaphorin), respectively. There is no research in the literature regarding the interactions of these proteins with Slug, and it seems to be open for further research.

## Conclusions

The results of this study supported the argument that, at molecular level, Skip N2 pathogenesis has different molecular background and regulation of SLUG expression may orchestrate the process, including EMT and the crosstalk between tumor and its microenvironment. TP63 had a strong correlation with SLUG expression in the bioinformatics analyses, which might influence EMT, CIP and/or apoptotic regulation of the tumor cells, that urges further investigations.

## Data Availability

Data and materials are available upon request.
